# Photo-oxidative killing of human colonic cancer cells using indocyanine green and infrared light

**DOI:** 10.1038/sj.bjc.6690363

**Published:** 1999-05-01

**Authors:** W Bäumler, C Abels, S Karrer, T Weiß, H Messmann, M Landthaler, R-M Szeimies

**Affiliations:** Departments of Dermatology, Internal Medicine I, and Surgery, University of Regensburg, 93042 Regensburg, Germany

**Keywords:** HT-29 cells, ICG, singlet oxygen, lipid peroxidation

## Abstract

Despite of the approval of Photofrin® in various countries, chemically defined sensitizers for photodynamic therapy (PDT) are still needed for the absorption of light in the infrared spectrum, which provides a maximal penetration of light into tissue. Therefore, both the efficacy and the mechanism of action of the clinically approved dye indocyanine green (ICG) and laser irradiation were investigated in vitro. For the investigation of phototoxic effects, HT-29 cells were incubated 24 h prior to irradiation by using different concentrations of ICG (10–500 μM). In each experiment, cells were irradiated using a continuous wave (cw)-diode laser (λ_ex_ = 805 nm, 30 J cm^−2^, 40 mW cm^−2^). After laser irradiation, cell viability of dark control and of cells incubated with 500 μM ICG was 1.27 ± 0.11 or 0.28 ± 0.05 respectively. Using 100 μM ICG and D_2_O, cell viability was further decreased from 0.46 ± 0.03 (H_2_O) to 0.11 ± 0.01 (D_2_O). Using D_2_O and 100 μM ICG, the concentration of malondialdehyde, a marker of lipid peroxidation, increased from 0.89 ± 0.10 nmol 10^−6^ cells to 11.14 ± 0.11 nmol 10^−6^ cells. Using 100 μM ICG and laser irradiation sodium azide or histidine (50 mM), quenchers of singlet oxygen reduced the cell killing significantly. In contrast, when using mannitol, a quencher of superoxide anion and hydroxyl radical, cell killing was not inhibited. According to the present results, photoactivated ICG seems to kill colonic cancer cells due to the generation of singlet oxygen and the subsequent formation of lipid peroxides. Therefore, ICG might present a promising photosensitizer for PDT; first clinical results confirm these findings. © 1999 Cancer Research Campaign


*British Journal of Cancer* (1999) **80**(3/4), 360–363
© 1999 Cancer Research Campaign
Article no. bjoc.1998.0363

### Photo-oxidative killing of human colonic cancer cells using indocyanine green and infrared light

**W Bäumler[1], C Abels[1], S Karrer, T Weiß[3], H Messmann[2], M Landthaler[1] and R-M Szeimies[1]**

Departments of [1]Dermatology, [2]Internal Medicine I and [3]Surgery, University of Regensburg, 93042 Regensburg, Germany

**Summary** Despite of the approval of Photofrin® in various countries, chemically defined sensitizers for photodynamic therapy (PDT) are still needed for the absorption of light in the infrared spectrum, which provides a maximal penetration of light into tissue. Therefore, both the efficacy and the mechanism of action of the clinically approved dye indocyanine green (ICG) and laser irradiation were investigated in vitro. For the investigation of phototoxic effects, HT-29 cells were incubated 24 h prior to irradiation by using different concentrations of ICG (10–500 μM). In each experiment, cells were irradiated using a continuous wave (cw)-diode laser ($\lambda_{ex}$ = 805 nm, 30 J cm$^{-2}$, 40 mW cm$^{-2}$). After laser irradiation, cell viability of dark control and of cells incubated with 500 μM ICG was 1.27 ± 0.11 or 0.28 ± 0.05 respectively. Using 100 μM ICG and $D_2O$, cell viability was further decreased from 0.46 ± 0.03 ($H_2O$) to 0.11 ± 0.01 ($D_2O$). Using $D_2O$ and 100 μM ICG, the concentration of malondialdehyde, a marker of lipid peroxidation, increased from 0.89 ± 0.10 nmol 10$^{-6}$ cells to 11.14 ± 0.11 nmol 10$^{-6}$ cells. Using 100 μM ICG and laser irradiation sodium azide or histidine (50 mM), quenchers of singlet oxygen reduced the cell killing significantly. In contrast, when using mannitol, a quencher of superoxide anion and hydroxyl radical, cell killing was not inhibited. According to the present results, photoactivated ICG seems to kill colonic cancer cells due to the generation of singlet oxygen and the subsequent formation of lipid peroxides. Therefore, ICG might present a promising photosensitizer for PDT; first clinical results confirm these findings.

**Keywords:** HT-29 cells; ICG; singlet oxygen; lipid peroxidation

Indocyanine green (ICG), which was approved by the United States Food and Drug Administration in 1956, is widely applied in medical diagnosis (Fox et al, 1956; Flower and Hochheimer, 1976; Moneta et al, 1987).

ICG shows a low incidence of adverse reactions (Hope-Ross et al, 1994) and has been intensively studied regarding its pharmacokinetics (Paumgartner et al, 1970). The absorption spectrum of this watersoluble, anionic tricarbocyanine dye exhibits a strong absorption band around 800 nm coinciding with the emission wavelength of a diode-laser (805 nm, see Figure 1).

For the evaluation of ICG as a possible photosensitizer, various photophysical parameters of ICG were determined by us in cooperation with the Department of Physics (University of Regensburg, Germany). The yields of triplet formation $\varphi_{S1-T1}$, by $S_1$–$T_1$ intersystem-crossing are 14% (in water, $H_2O$), 16% (in methanol), 17% (in dimethypl sulphoxide, DMSO) and 11% (in aqueous albumin solution) (Reindl et al, 1997). In comparison to HPD (in methanol, 82%) and aluminium phthalocyanine (in water, 38%) (Grossweiner, 1994) the triplet yields of ICG appear low, but the values of ICG might be high enough to act as a photosensitizer for photodynamic therapy (PDT).

First, in vitro experiments using ICG and normal keratinocytes (Fickweiler et al, 1997) showed phototoxic effects. However, the mechanism of action – photo-oxidative versus photothermal cell killing – remained unclear and was investigated again using carcinoma cells (colon cancer, HT-29).

*Received 19 June 1998*
*Revised 16 November 1998*
*Accepted 17 November 1998*

*Correspondence to:* R-M Szeimies

### MATERIALS AND METHODS

### Cell culture and dye preparation

The immortalized human colon carcinoma cell line HT-29 (Fogh and Trempe, 1975) was maintained in Dulbecco's modified Eagle's medium (DMEM; Sigma Chemie, Deisenhofen, Germany) and supplemented with 10% fetal calf serum (FCS; Sigma Chemie), 1% L-glutamine and 1% streptomycin–penicillin (Gibco, Eggenstein, Germany) in a humidified atmosphere containing 8% carbon dioxide at 37°C. Cells were washed with phosphate-buffered saline (PBS; Biochrom, Berlin, Germany) and harvested by a 10 min treatment with 0.05% trypsin/0.02% EDTA (Gibco) in PBS. For in vitro assays, ICG (molar mass of the ICG sodium iodide: 924.9 g mol$^{-1}$; PULSION Medizintechnik, München, Germany) was dissolved in growth medium at concentrations of 10, 50, 100 and 500 μM.

### Treatment protocol and phototoxicity assay

HT-29 cells were seeded at equal concentrations (15 × 10$^3$ cells in 100 μl medium per well) into 96-well microtitre plates (Costar, Tecnomara, Fernwald, Germany). After overnight cell attachment, medium was replaced by 100 μl ICG solution at various concentrations (10, 50, 100 or 500 μM). Following an incubation time of 24 h at 37°C, supernatants were removed. In order to eliminate remaining dye, cells were carefully washed with medium and covered with 100 μl of drug-free medium prior to irradiation. The cells for control experiments were processed in the same way.

Irradiation of cells incubated with ICG was performed using a continuous wave (cw)-diode-laser emitting light at 805 nm (Opto Power Corp., CA, USA) with a maximum optical output power of 15 W. The laser light was coupled into a monocore fibre with 1.5 mm diameter and distributed to a flat homogeneous area (150 $cm^2$, Ø 14 cm) sufficient to cover a 96-well microtitre plate by a biconvex lens with a fluence of 40 mW $cm^{-2}$. The fluence used was 30 J $cm^{-2}$. Measuring the temperature of supernatants by a thermocouple immediately after irradiation ensured that no hyperthermic conditions were imposed by this irradiation arrangement.

Following irradiation, cells were maintained under normal culture conditions for a further 24 h. Viability of cells after ICG and/or light treatment was assessed by means of the 3-4,5-dimethylthiazol-2,5-diphenyl tetrazolium bromide (MTT) assay (for details see McHale and McHale, 1988). The ratio of the optical densities of treated cells to untreated cells, which served as control, was referred to as cell viability (CV).

### Effect of sodium azide, histidine and mannitol on cell killing

Sodium azide, histidine or mannitol effective physical quencher of reactive oxygen species (Moan et al, 1979; Agarwal et al, 1991; Hampton et al, 1993; Zaidi et al, 1993) were added to the cell culture 60 min prior to irradiation. HT-29 cells incubated with 100 μM ICG for 24 h were irradiated (30 J $cm^{-2}$) as described above but in presence of sodium azide, histidine or mannitol (Merck) at a concentration of 50 mM in PBS. CV was assessed as described above.

### Effect of $D_2O$ on cell killing

Cell killing effects are enhanced using $D_2O$ in vitro (Lin et al, 1991). $D_2O$ was added 60 min prior to irradiation. HT-29 cells incubated with 100 μM ICG for 24 h were irradiated (30 J $cm^{-2}$) as described above but in presence of $D_2O$. CV was assessed as described above.

### Lipid peroxidation assay

Unsaturated lipids are important targets of membrane photo-oxidation. Malondialdehyde (MDA), an ultimate marker of lipid peroxidation, can be measured by the fluorometric thiobarbituric acid (TBA) assay (Perret et al, 1994). After irradiation the cells were homogenized using ultrasound and centrifuged for 15 min. The suspension was mixed with phosphortungstic acid (10% in $H_2O$), sodium dodecyl sulphate (7% in $H_2O$), hydrochloric acid (0.1 M), thiobarbituric acid (0.5% in $H_2O$) and shaken for 60 min at a temperature of 95°C. After cooling down, the MDA–TBA complex was extracted by *n*-butanol. The quantification of the MDA–TBA complex was performed by measuring its fluorescence at 553 nm; a MDA standard solution (0–3 nmol $ml^{-1}$) served as control.

### Data analysis and statistics

Each individual experiment was carried out at least in triplicate. For the MTT assay, at least ten individual wells were plated with cells treated in an identical matter with their mean optical density used for data analysis. The effects of the different treatment modalities were expressed as CV of treated cells compared with untreated controls. Differences were tested for statistical significance using the two-sided *t*-test. All primary data are presented as mean and standard deviations of the mean.

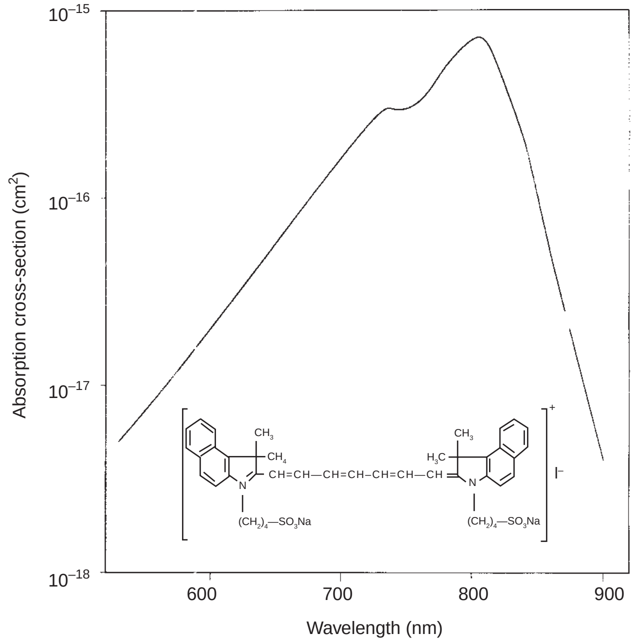

**Figure 1** Chemical structure and absorption spectrum of ICG sodium iodide

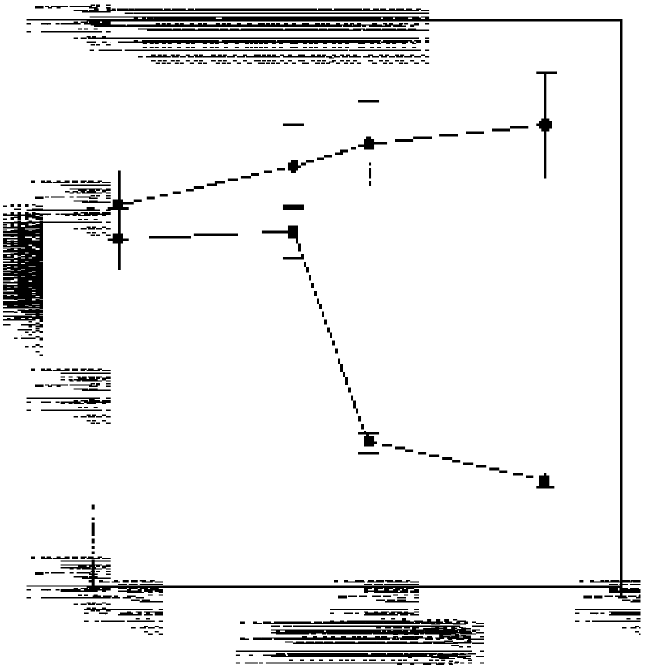

**Figure 2** Concentration finding studies with (■) and without (●) irradiation by a cw-diode laser (805 nm, 40 mW $cm^{-2}$, 30 J $cm^{-2}$). Cell viability of colonic cancer cells HT-29 following incubation for 24 h using different concentrations of ICG. Each point is the mean of at least three determinations ± standard deviation

### RESULTS

### ICG phototoxicity in vitro

The experiments revealed no dark toxicity in any ICG concentration used. Irradiation used on its own did not result in a significant decrease of CV. Viability assays 24 h after irradiation exhibited a

*© Cancer Research Campaign 1999*

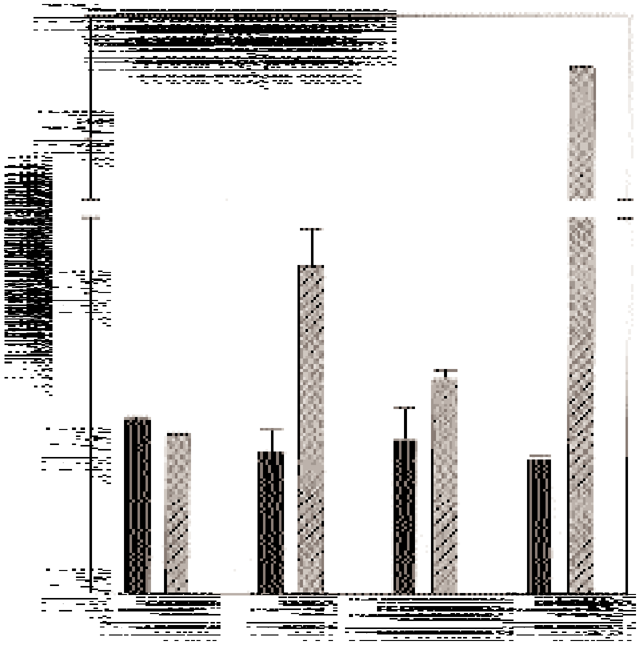

**Figure 3** Effects of histidine (50 mM) and $D_2O$ on ICG-incubated HT-29 cells (100 μM ICG) with and without light irradiation (diode-laser, 805 nm, 40 mW cm$^{-2}$). The black bars represent MDA concentration using ICG alone and ICG in combination with histidine or $D_2O$, the grey bars represent the MDA concentration using ICG alone and ICG in combination with histidine or $D_2O$ following irradiation. Each bar is the mean of 3 determinations ± standard deviation

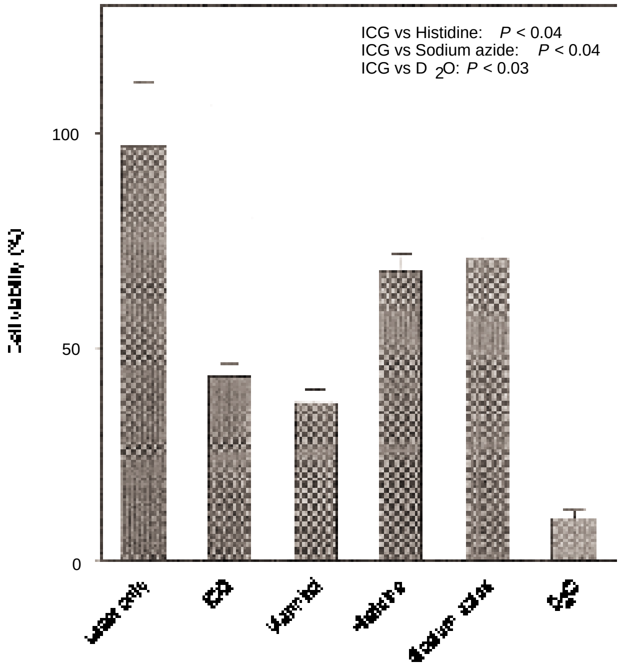

**Figure 4** Effect of sodium azide (50 mM), histidine (50 mM) mannitol (50 mM) and $D_2O$ on ICG (100 μM) mediaed cell killing determined by the MTT assay (incubation time 24 h, total light dose 30 J cm$^{-2}$). Each bar is the mean of three determinations ± standard deviation behaviour inversely proportional to the used ICG concentrations as illustrated in Figure 2. Starting with a concentration of 10 μM ICG, the CV decreased according to an increasing ICG concentration by using a light dose of 30 J cm$^{-2}$. CV of dark control and cells incubated with 500 μM ICG and irradiated with 30 J cm$^{-2}$ was 1.27 ± 0.14 and 0.28 ± 0.02 respectively.

### Lipid peroxidation

MDA, a marker for lipid peroxidation, increased from 0.93 ± 0.16 nmol 10$^{-6}$ cells to 2.17 ± 0.26 nmol 10$^{-6}$ cells after light irradiation (30 J cm$^{-2}$) of HT-29 cells incubated with 100 μM ICG for 24 h (Figure 3). Using histidine, the amount of MDA was 1.42 ± 0.05 nmol 10$^{-6}$ cells with light and 1.01 ± 0.24 nmol 10$^{-6}$ cells without light. Adding $D_2O$, however, dramatically increased MDA from 0.89 ± 0.10 nmol 10$^{-6}$ cells to 11.14 ± 0.11 nmol 10$^{-6}$ cells.

### Effects of different quenchers and $D_2O$ on cell killing

Figure 4 shows the effect of incubation with ICG (100 μM) and quenchers (50 mM) or $D_2O$ prior to light irradiation (30 J cm$^{-2}$). Using ICG and laser irradiation only, CV was 0.46 ± 0.03. The cell killing of ICG was reduced in the presence of sodium azide indicated by an increase of the CV from 0.46 ± 0.03 to 0.65 ± 0.04. In the presence of histidine, CV increased from 0.46 ± 0.03 to 0.64 ± 0.05. By adding 50 mM mannitol to the cells, CV did not change significantly, 0.46 ± 0.03 and 0.40 ± 0.04 respectively, whereas the addition of $D_2O$ revealed a dramatic decrease of CV from 0.46 ± 0.03 to 0.11 ± 0.06.

### DISCUSSION

The present results show that human colon carcinoma cells can be effectively destroyed by photoactivated ICG. Irradiation with a low energy density (30 J cm$^{-2}$) significantly decreased the cell viability. Other experiments in vitro using different sensitizers and a different setup, yielded a similar cell toxicity (Ma et al, 1994; Jori, 1996; Rossi et al, 1996; Fickweiler et al, 1997). However, ICG concentrations up to 500 μM did not exhibit a dark toxicity. The photosensitizers used, e.g. haematoporphyrin (Bachor et al, 1995), methylene blue (Wainwright et al, 1997) or porphycenes (Bäumler et al, 1995), usually resulted in an increasing dark toxicity with increasing sensitizer concentrations in vitro.

Evaluating the mechanism of action regarding the cell killing by a photosensitizer, specific experiments should be performed since the absorbed energy of the laser light by the molecule of the used photosensitizer can reach the ground state by either radiative (fluorescence) or non-radiative decay. In the non-radiative decay the absorbed energy of the molecule is converted either to heat (internal conversion) and transferred to other molecules (photo-oxidation, type I), or transferred to molecular oxygen (photooxidation, type II) via triplet-state.

Photothermal effects due to internal conversion damage cells by an increase of the intracellular temperature, shown by the use of ICG for photocoagulation or tissue welding (Decoste et al, 1992; Reichel et al, 1994). If photothermal effects are responsible for cell killing by ICG, quenchers like sodium azide or histidine should fail to reduce ICG-induced phototoxicity.

In order to investigate whether photo-oxidation of type I or type II are the predominant mechanisms of action mannitol, a quencher of superoxide anion, hydroxyle radical (type I) and histidine or sodium azide, quenchers of singlet oxygen (type II) were used (Agarwal et al, 1991; Zaidi et al, 1993; Wlaschek et al, 1995). The results seen in Figure 4 show that mannitol did not protect HT-29 cells against photoactivated ICG, whereas the quenchers sodium

© Cancer Research Campaign 1999

azide or histidine significantly reduced the cell killing. Thus, in the present in vitro experiments, the killing of HT-29 cells by ICG and laser irradiation seem to be mediated mainly by singlet oxygen. Moreover, adding $D_2O$ to the culture medium, photo-oxidative cell killing was further enhanced due to a prolonged lifetime of singlet oxygen in the presence of $D_2O$, about 40 μs versus 3 μs in $H_2O$ (Matheson et al, 1978). In addition, the increase of lipid peroxides in vitro as measured by means of the fluorometric TBA assay (Perret et al, 1994), confirms that photo-oxidation of HT-29 cell structures is responsible, to some extent, for the cell killing in the chosen experimental setup (Figure 3). The increase of MDA adding $D_2O$ to the cell culture medium may be a hint for oxidative destruction of membranes due to the presence of singlet oxygen and its prolonged lifetime in $D_2O$.

In contrast to many other sensitizers, e.g. porphyrine derivatives, which are photoactivated by red light ranging from 600 to 700 nm, ICG exhibits a high absorption cross-section in the infrared region at 805 nm. Infrared light penetrates deeper into tissue than red light (Anderson et al, 1981), presenting an outstanding advantage, if ICG acts as a photosensitizer in vivo.

Due to the clinical approval of ICG, the successful treatment of Kaposi's sarcomas was already shown in a first pilot study, using a different experimental setup (Abels et al, 1998). Further investigations regarding the exact mechanism of action in vivo – photodynamic versus photothermal – will be performed.

### ACKNOWLEDGEMENTS

We acknowledge technical assistance of P Weiderer.

### REFERENCES

Abels C, Karrer S, Bäumler W, Goetz AE, Landthaler M and Szeimies RM (1998) Indocyanine green and laser light for the treatment of AIDS-associated cutaneous Kaposi's sarcoma. *Br J Cancer* **77**: 1021–1024.

Agarwal R, Athar M, Urban SA, Bickers DR and Mukhtar H (1991) Involvement of singlet oxygen in chloroaluminum phthalocyanine tetrasulfonate-mediated photoenhancement of lipid peroxidation in rat epidermal microsomes. *Cancer Lett* **56**: 125–129

Anderson R, Hu J and Parrish J (1981) Optical radiation transfer into human skin. In *Bioengeneering and the Skin*, Marks R, Payne P (eds), pp. 253–265. Lancaster MTP, Boston, USA

Bachor R, Reich E, Miller K, Rück A and Hautmann R (1995) Photodynamic efficiency of liposome-administered tetramethyl hematoporphyrin in two human bladder cancer cell lines. *Urol Res* **23**: 151–156

Bäumler W, Szeimies RM, Abels C, Karrer S and Landthaler M (1995) [9]acetoxy-2,7,12,17-tetrakis-(β-methoxyethyl)-porphycene (ATMPn), a novel photosensitizer for photodynamic therapy – dose-dependent effects in vitro. In *Photochemotherapy: Photodynamic Therapy and Other Modalities*, Ehrenberg B, Jori G, Moan J, (eds). *Proc. SPIE* **2625**: 315–318

Decoste S, Farinelli W, Flotte T and Anderson R (1992) Dye-enhanced laser welding for skin closure. *Laser Surg Med* **12**: 25–32

Fickweiler S, Szeimies R-M, Bäumler W, Steinbach P, Karrer S, Goetz A-E, Abels C, Hofstädter F and Landthaler M (1997) Indocyanine green: intracellular uptake and phototherapeutic effects. *J Photochem Photobiol* B **38**: 178–183

Flower R and Hochheimer B (1976) Indocyanine green dye fluorescence and infrared absorption choroidal angiography performed simultaneously with fluorescein angiography. *Johns Hopkins Med J* **138**: 33–42

Fogh J and Trempe G (1975) In *Human Tumor Cells In Vitro* Fogh J (ed), pp. 115–159. Plenum Press: New York

Fox I, Brooker G, Heseltine D, Essex H and Wood E (1956) New dyes for continuous recording of dilution curves in whole blood independent of variations in blood oxygen saturation. *Am J Physiol* **187**: 599

Grossweiner L (1994) *Science of Phototherapy*. CRC Press: London

Hampton J, Skalkos D, Taylor P and Selman S (1993) Iminium salt of copper benzochlorin (CDS1), a novel photosensitizer for photodynamic therapy: mechanism of cell killing. *Photochem Photobiol* **58**: 100–105

Hope-Ross M, Yannuzzi L, Gragoudas E, Guyer D, Slakter J, Sorenson J, Krupsky S, Orlock D and Puliafito C (1994) Adverse reactions due to indocyanine green. *Ophthalmology* **101**: 529–533

Jori G (1996) Tumour photosensitizers: approaches to enhance the selectivity and efficiency of photodynamic therapy. *J Photochem Photobiol B* **36**: 87–93

Lin CW, Shulok JR, Wong YK, Schanbacher CF, Cincotta L and Foley JW (1991) Photosensitization, uptake and retention of phenoxazine nile blue derivatives in human bladder carcinoma cells. *Cancer Res* **51**: 1109–1116

McHale A and McHale D (1988) Use of a tetrazolium based colorimetric assay in assessing photoradiation therapy in vitro. *Cancer Lett* **41**: 315–321

Ma L, Moan J and Berg K (1994) Evaluation of a new photosensitizer, meso-tetra-hydroxyphenyl-chlorin, for use in photodynamic therapy: a comparison of its photobiological properties with those of two other photosensitizers. *Int J Cancer* **57**: 883–888

Matheson IB, Lee J and King AD (1978) The lifetime of singlet oxygen in heavy water. *Chem Phys Lett* **55**: 49

Moan J, Pettersen E and Christensen T (1979) The mechanism of photodynamic inactivation of human cells in vitro in the presence of haematoporphyrin. *Br J Cancer* **39**: 398–407

Moneta G, Brülisauer M, Jäger K and Bollinger A (1987) Infrared fluorescence videomicroscopy of skin capillaries with indocyanine green. *Int J Microcirc Clin Exp* **6**: 25–34

Paumgartner G, Probst P, Kraines R and Leevy C (1970) Kinetics of indocyanine green removal from the blood. *NY Acad Sci* **170**: 134–170

Perret C, Foultier MT, Vonarx-Coinsman V, Quancard O, Combre A and Patrice T (1994) Malondialdehyde dosimetry in laser-irradiated tissues sensitized by hematoporphyrin derivative. *J Pharmacol Exp Ther* **269**: 787–791

Reichel E, Puliafito C, Duker J and Guyer D (1994) Indocyanine green dye-enhanced diode laser photocoagulation of poorly defined subfoveal choroidal neovascularization. *Ophthalm Surg* **25**: 195–201

Reindl S, Penzkofer A, Gong S-H, Landthaler M, Szeimies R-M, Abels C and Bäumler W (1997) Quantum yield of triplet formation for indocyanine green. *J Photochem Photobiol A* **105**: 65–68

Rossi FM, Campbell DL, Pottier RH, Kennedy JC and Dickson EF (1996) In vitro studies on the potential use of 5-aminolaevulinic acid-mediated photodynamic therapy for gynaecological tumours. *Br J Cancer* **74**: 881–887

Wainwright M, Phoenix DA, Rice L, Burrow SM and Waring J (1997) Increased cytotoxicity and phototoxicity in the methylene blue series via chromophore methylation *J Photochem Photobiol B* **40**: 233–239

Wlaschek M, Briviba K, Stricklin GP, Sies H and Scharffetter-Kochanek K (1995) Singlet oxygen may mediate the ultraviolet A-induced synthesis of interstitial collagenase. *J Invest Dermatol* **104**: 194–198

Zaidi SI, Agarwal R, Eichler G, Rihter BD, Kenney ME and Mukhtar H (1993) Photodynamic effects of new silicon phthalocyanines: in vitro studies utilizing rat hepatic microsomes and human erythrocyte ghosts as model membrane sources. *Photochem Photobiol* **58**: 204–210

© Cancer Research Campaign 1999
